# N^6^-Methyladenosine-regulated LINC00675 suppress the proliferation, migration and invasion of breast cancer cells via inhibiting miR-513b-5p

**DOI:** 10.1080/21655979.2021.2001905

**Published:** 2021-12-01

**Authors:** Shenglan Fan, Liping Wang

**Affiliations:** a Department of Breast Surgery, Hubei Cancer Hospital, Tongji Medical College, Huazhong University of Science and Technology and Hubei Provincial Clinical Research Center for Breast Cancer, Wuhan, China; b Department of Gynecological Oncology, Hubei Cancer Hospital, Tongji Medical College, Huazhong University of Science and Technology, Wuhan, China

**Keywords:** miR-513b-5p, ceRNA, m^6^Amethylation, METTL3

## Abstract

Breast cancer (BC) is the most common cancer among women. LINC00675 and miR-513b-5p has been reported to be abnormally expressed in multiple types of cancers and modulate malignant phenotypes of cancer cells. However, to date, the functional role and underlying regulatory mechanism of LINC00675 and miR-513b-5p in BC remains largely unknown. Here, we found that LINC00675 was significantly downregulated in BC tissues and cell lines. Decrease of LINC00675 expression associated with higher tumor grade, lymphovascular invasion and shorter survival in BC patients. Functional experiments demonstrated that overexpression of LINC00675 suppressed BC cell proliferation, migration and invasion, whereas depletion of LINC00675 exerted opposite effects. Mechanistically, LINC00675 functioned as a competing endogenous RNA (ceRNA) to interact with miR-513b-5p and suppress its expression. Moreover, METTL3 increased the m^6^A methylation of LINC00675, which enhanced the association between LINC00675 and miR-513b-5p. Collectively, the central findings of our study suggest that LINC00675 represses BC progression through the inhibition of miR-513b-5p in a m^6^A-dependent manner.

## Introduction

Breast cancer (BC) is the most commonly diagnosed cancer among women, and it is a serious threat for women’s health worldwide [1]. Even through the great advances made in early diagnosis or treatment, including surgery, chemotherapy, radiotherapy and targeted therapy, resulted in substantial improvement in the therapeutic effects, the incidence and the mortality rate of BC remains high [2,3]. Therefore, it is necessary to reveal the mechanisms of BC initiation and development so that novel effective molecules and targets for diagnosis and treatment for BC could be developed.

The advancement of high-throughput sequencing technologies has provided new insights into the importance of long non-coding RNAs (lncRNAs) in gene regulation. LncRNAs are a class of non-protein-coding RNA transcripts that are longer than 200 nt. Increasing evidence has suggested that lncRNAs are involved in physiological and pathological processes at the epigenetic, transcriptional, post-transcriptional, translational, and post-translational levels. LncRNAs act as key regulators in cancer, which regulate oncogenes and tumor-suppressor genes to impact tumorigenesis, metastasis, drug resistance, angiogenesis and prognosis via interacting with DNA, mRNA, microRNA, and proteins [4–6]. For instance, lncRNA HOTAIR interacts with polycomb repressive complex 2 (PRC2) and plays a pivotal role in the H3K27 methylation of many genes involved in cellular death, motility, and cycle progress [7]. The involvement of lncRNAs in BC development has been explored. Overexpression of lncRNA BCRT1 correlates with poor prognosis of BC patients. LncRNA BCRT1 acts as a competing endogenous RNA (ceRNA) to competitively bind with miR-1303 and prevent the degradation of its target gene PTBP3, resulting in BC growth and metastasis *in vitro* and *in vivo* [8]. LncRNA FGF13-AS1 expression is decreased in BC tissue. FGF13-AS1 functions as a tumor suppressor to inhibit BC cell proliferation, migration, and invasion by impairing glycolysis and stemness properties. FGF13-AS1 promotes the degradation of c-Myc mRNA via attenuating the interaction between IGF2BPs and c-Myc mRNA [9]. LINC00675 locates in chromosome 17 and has been reported to be abnormally expressed in multiple types of cancers, such as pancreatic ductal adenocarcinoma, gastric cancer, glioma, cervical cancer, esophageal squamous cell carcinoma, and prostate cancer [10–15]. However, there is no previous report about the functional role and underlying mechanism of LINC00675 in BC cells.

N^6^-Methyladenosine (m^6^A) modification is the most abundant eukaryotic RNA. m^6^A modification regulates essential biological processes of the target RNAs, including RNA splicing, decay, nuclear export, translation, microRNA processing, and the ceRNA activity of lncRNA [16,17]. Emerging evidence demonstrated that lncRNAs-regulated m^6^A modification of target RNAs is involved in tumor initiation and development [18,19]. However, the functional relevance between LINC00675 and m^6^A modification remains elusive.

In the present study, we sought to investigate the expression pattern and functions of LINC00675 in BC. Moreover, we provided mechanistic insights into the regulation of miR-513b-5p by LINC00675 in a m^6^A-dependent manner.

## Materials and methods

### Specimens

Seventy-four patients who were diagnosed with BC and underwent surgical resection at Hubei Cancer Hospital between 2015 and 2017 were randomly enrolled in this research. Fresh specimens of paired BC and adjacent non-tumor tissues were obtained from these 74 patients. All patients did not receive any treatment before surgery and signed the informed consent. Patients with other cancer or receiving chemotherapy, radiotherapy, or targeted therapy before surgery were excluded. All tissue samples were frozen in liquid nitrogen immediately and stored in −80°C until used. This study was approved in 2015 by the Ethics Committee of Hubei Cancer Hospital, followed the clinical research guidelines (ethics approval number: no. 2019–04-212), and complied the Declaration of Helsinki.

### Cell culture

Five breast cancer cell lines (MCF7, T47D, BT474, MDA231, and BT549) and one normal breast cell line (MCF10A) were obtained from the Cell Bank of the Chinese Academy of Sciences (Shanghai, China). All cells were cultured in Dulbecco’s modified Eagle’s medium (DMEM) supplemented with 10% fetal bovine serum at 37°C in an atmosphere containing 5% CO_2_.

### Transfection

The mutant LINC00675 was constructed using TaKaRa MutanBEST Kit. The wild-type and mutant LINC00675 were cloned into the overexpression plasmid (pcDNA3.1), which was constructed by Genepharma Company (Shanghai, China). miR-513b-5p mimics and negative control were purchased from Genepharma (Shanghai, China). LINC00675 and METTL3 siRNAs and negative control were synthesized and purchased from Genepharma (Shanghai, China). The Lipofectamine 2000 kit (Invitrogen) was used for cell transfection according to the manufacturer’s instructions. Forty-eight hours after transfection, cells were subjected for further experiments. The target sequence of siRNAs was listed as follow: LINC00675 siRNA#1 (KD1): CTGGGACTTCTTCATCTAC, LINC00675 siRNA#2 (KD2): GCTGCAATACTGAGGCTTT, siMETTL3: GCTACAGATCCTGAGTTAG.

### Subcellular fractionation

Nuclear and cytoplasmic RNA was isolated using the PARIS Kit (Life Technologies, USA) according to the manufacturer’s instructions.

### Cell counting kit-8 (CCK-8) and colony formation assay

3 × 10^3^ cells per well were seeded into a 96-well plate and incubated for 1–4 days. After that, 10 μl CCK-8 solution was added into each well. The plate was incubated for another 1 h. The optical density (OD) value of each well was measured. For colony formation assay, 2× 10^3^ cells per well were seeded into a 6-well plate and incubated for 7 days. Cells were fixed with 4% paraformaldehyde and stained with 0.1% crystal violet and then counted

### Transwell assay

Transwell assays were performed to detect cell migration and invasion. For the migration detection, cells in serum-free media were seeded into the upper Transwell chambers (Corning). For the invasion detection, cells in serum-free media were seeded into the upper Matrigel-coated Transwell chambers (BD Bioscience). The DMEM with 10% FBS was added to the lower chamber. After 24 h incubation, cells in the lower chamber were fixed with 4% paraformaldehyde and stained with 0.1% crystal violet. The cells were counted in 10 random field under microscope.

### RNA extraction and quantitative real-time PCR (qRT-PCR)

Total RNA from cells or tissues was isolated using Trizol reagent (Invitrogen) according to the standard protocol. First-strand cDNA was synthesized using the EasyScript® One-Step gDNA Removal and cDNA Synthesis SuperMix (Transgen, Beijing). qRT-PCR was carried out in the Lightcycler 96 (Roche) using SYBR® Green (Takara, Dalian, China). The relative expression of RNAs was calculated using 2^−ΔΔCT^ method. The sequence of gene-specific primers was listed as follow: LINC00675-forward: AAGGAGATGCCCTTCCTTTAC, LINC00675-reverse: GGACATCCTCGTGAGTACTTTG, METTL3-forward: CACTGATGCTGTGTCCATCT, METTL3-reverse: CTTGTAGGAGACCTCGCTTTAC.

### RNA immunoprecipitation (RIP)

RIP assay was used to determine the association of LINC00675 and METTL3 or AGO2. Antibodies used for the RIP assay included anti-AGO2 (Millipore), METTL3 (Abcam) and control IgG (Millipore, USA). In brief, cells were lysed in RIP lysis buffer, and then incubated with indicated antibody and Magna beads. After wash, the coprecipitated RNAs were purified and then subjected to qRT-PCR analysis.

### MS2bs (MS2-binding protein)-MS2bp (MS2-binding sequences)-based RIP (MS2-RIP)

MS2-RIP assay was used to determine the association of LINC00675 and miR-513b-5p. MS2-RIP was performed as previous studies described [20,21]. In brief, cells were co-transfected with pcDNA3.1-MS2, pcDNA3.1-MS2-LINC00675, or pcDNA3.1-MS2-LINC00675-mut and pMS2-GFP. After 48 hrs, cells were used to perform RNA immunoprecipitation (RIP) experiments using a GFP antibody (Roche) and the Magna RIP™ RNA-Binding Protein Immunoprecipitation Kit (Millipore, Bedford, MA) according to the manufacturer’s instructions. Antibodies used for the MS2-RIP assay included anti-GFP (Millipore) and control IgG (Millipore, USA). The coprecipitated RNAs were purified and then subjected to qRT-PCR analysis.

### Methylated RNA immunoprecipitation (MeRIP)

MeRIP experiment was carried out to detect the m^6^A level of LINC00675 using Magna MeRIP™ m^6^A Kit (Millipore) according to the manufacturer’s instructions. In brief, total RNAs were first extracted from cells. RNAs were treated with DNase using TURBO DNA-free^TM^ Kit (Thermo) to avoid DNA contaminations. RNA concentration was adjusted to 1 μg/μl with nuclease-free water. RNA was chemically fragmented into ~100nt size and fragmented RNA was then incubated with m^6^A antibody for immunoprecipitation according to the standard protocol of Magna MeRIP™ m^6^A Kit (Merck Millipore).

### RNA pull-down assay

LINC00675 or mutant LINC00675 (LINC00675-mut) were *in vitro* transcribed, respectively, and biotin-labeled with the Biotin RNA Labeling Mix (Roche) and T7 RNA polymerase (Roche), treated with RNase-free DNase I (Roche), and purified with a RNeasy Mini Kit (Qiagen). Cell lysates were incubated with purified biotinylated transcripts for 1 hour; complexes were isolated with streptavidin agarose beads (Invitrogen). The miR-513b-5p pulled down by LINC00675 was detected by qRT-PCR analysis.

### Luciferase reporter assay

Wild-type or mutant LINC00675 were cloned into luciferase reporter plasmid pmirGLO. pmirGLO, pmirGLO-LINC00675 or pmirGLO-LINC00675-mut was cotransfected with miR-513b-5p mimics or miR-NC into cells using the Lipofectamine 2000 kit. 48 hours later, the relative luciferase activity was detected and normalized to Renilla luciferase activity.

### Statistical analysis

All experiments were performed with at least three biological replicates in triplicate. Data are expressed as the mean ± SD and analyzed by the SPSS software. Student t test (for two groups) or one-way ANOVA (for groups more than two) was used for the comparisons of different groups. Chi-square test was used to analyze the association between LINC00675 expression and clinicopathological features of BC patients. Kaplan-Meier method and log-rank test was performed for determine the relationship between LINC00675 expression and prognosis of BC patients. P less than 0.05 was considered statistically significant.

## Results

### Downregulation of LINC00675 is associated with poor prognosis of BC patients

**Figure 1.**
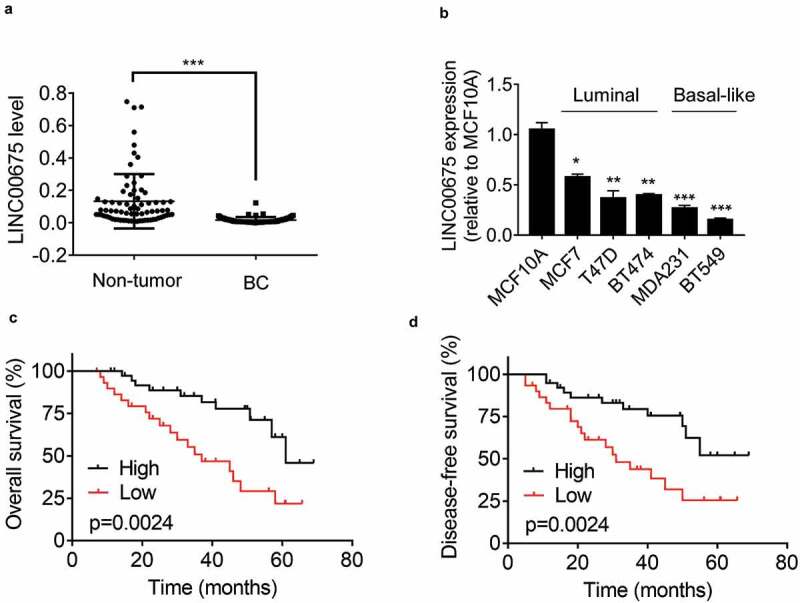
Downregulation of LINC00675 is associated with poor prognosis of BC patients

To determine the expression pattern of LINC00675 in BC, the LINC00675 levels in 74 pairs of BC and adjacent non-tumor tissues were detected using qRT-PCR. The results showed that the BC tissues expressed obviously lower LINC00675 expression than non-tumor tissues did (Figure 1(a)). Additionally, compared to normal breast cell line MCF10A, LINC00675 was significantly lower in BC cell lines, such as MCF7, T47D, BT474, MDA231, and BT549 (Figure 1(b)). LINC00675 was high in luminal cell lines and low in basal-like breast cancer (BLBC) cell lines. Among these BC cells, BT549 cells expressed the lowest level of LINC00675 expression, while MCF7 cells exhibited the highest LINC00675 expression. These two cell lines were used for overexpression and knockdown experiments, respectively.

(a) qRT-PCR analysis of LINC00675 levels in 74 pairs of BC tissues and nearby non-tumor tissues. ***p < 0.001. (b) qRT-PCR analysis of relative LINC00675 levels in normal breast cell line MCF10A and different BC cell lines. BLBC, basal-like breast cancer. (c) Kaplan-Meier analysis of the overall survival of BC patient with high and low LINC00675 (log rank test). The median of LINC00675 in BC tissues was used as cutoff. (d) Kaplan-Meier analysis of the disease-free survival of BC patient with high and low LINC00675 (log rank test). The median of LINC00675 in BC tissues was used as cutoff.

To reveal the clinical significance of LINC00675 expression in BC, we analyze the correlation between LINC00675 expression and clinicopathological features of BC patients. According to the median value of LINC00675 expression in BC tissues, enrolled BC patients were divided into high-expression and low-expression groups. The correlation analysis demonstrated that decreased LINC00675 expression was significantly associated with higher tumor grade and lymphovascular invasion (Table 1). Moreover, Kaplan-Meier survival analysis showed that low LINC00675 expression was correlated with poor overall survival of BC patients (Figure 1(c)). Together, these data suggest that LINC00675 may act as a tumor suppressive lncRNA in BC.

**Table 1.** The correlation analysis between LINC00675 expression and clinicopathologic features of 74 BC patients

Clinicopathological features	LINC00675 level		P value
	Low (n = 37)	High (n = 37)	
Age			0.816
≤45	20	19	
> 45	17	18	
Subtype			0.492
ER+/PR+	28	27	
HER2 positive	6	4	
TNBC	3	6	
Menopause			0.480
premenopause	23	20	
promenopause	14	17	
Tumor size			0.352
≤ 2 cm	17	21	
> 2 cm	20	16	
Tumor grade			0.002
I–II	17	30	
III	20	7	
Lymphovascular invasion			0.020
Negative	25	15	
Positive	12	22	

ER, estrogen receptor; PR, progesterone receptor; HER2, human epidermal growth factor receptor 2; TNBC, triple-negative breast cancer.

The median of LINC00675 in BC tissues was used as cutoff.

### LINC00675 acts as a tumor suppressor in BC cells

**Figure 2.**
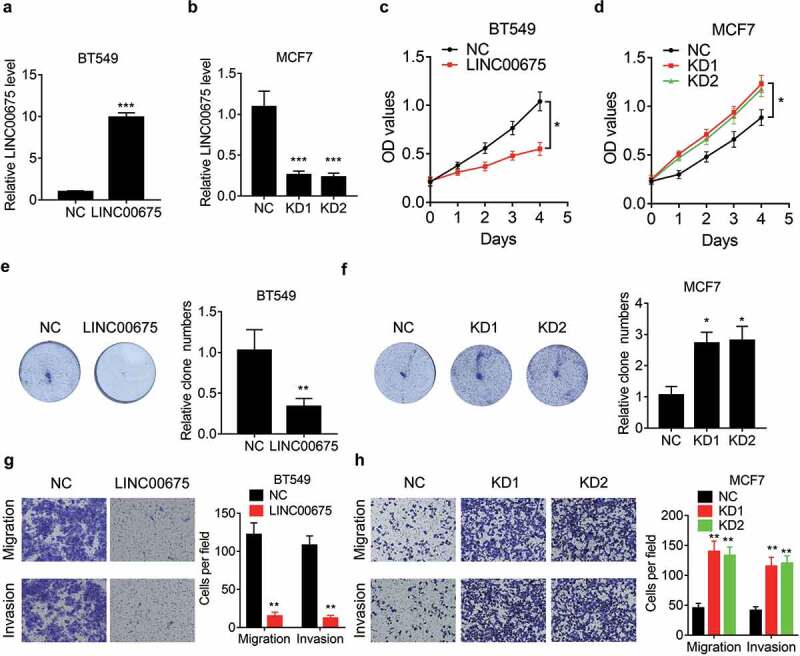
LINC00675 suppresses the proliferation, migration and invasion of BC cells

To elucidate the functional role of LINC00675 in BC cells, LINC00675 was overexpressed in BT549 cells, but knocked down in MCF7 cells. The overexpression and knockdown efficiency was validated using qRT-PCR (Figure 2(a,b)). We performed CCK-8 assay to evaluate the effect of LINC00675 on BC cell proliferation and found that overexpression of LINC00675 significantly suppressed the proliferation of BT549 cells (Figure 2(c)). Conversely, the proliferative ability was enhanced by transfection of LINC00675 siRNAs in MCF7 cells (Figure 2(d)). The results of colony formation assay further validated the suppressive effect of LINC00675 in BC cell proliferation (Figure 2(e, f)).

(a) The plasmid expressing nothing or LINC00675 was transfected into BT549 cells. After 48 hours, the expression of LINC00675 in indicated cells was detected using qRT-PCR. (b) The negative control or LINC00675 siRNAs were transfected into MCF7 cells. After 48 hours, the expression of LINC00675 of indicated cells was detected using qRT-PCR. (c) CCK-8 assay was performed in control and LINC00675 overexpressing BT549 cells. (d) CCK-8 assay was performed in control and LINC00675 silencing MCF7 cells. (e) The colony formation ability of control and LINC00675 overexpressing BT549 cells. The representative images and statistical results were shown. (f) The colony formation ability of control and LINC00675 silencing MCF7 cells. The representative images and statistical results were shown.G.Transwell migration assay and invasion assay was performed in control and LINC00675 overexpressing BT549 cells. The represent images (left) and statistical result (right) was shown. The cells were counted in ten random field under microscope. (h) Transwell migration assay and invasion assay was performed in control and LINC00675 silencing MCF7 cells. The represent images (left) and statistical result (right) was shown. The cells were counted in ten random field under microscope.*P < 0.05, **P < 0.01, ***P < 0.001.

We then evaluated the function of LINC00675 in BC cell migration and invasion. Transwell assay results indicated that ectopic expression of LINC00675 obviously attenuated (Figure 2(g)), while knockdown of LINC00675 enhanced, migration, and invasion in BC cells (Figure 2(h)), supporting a metastasis-suppressive role of LINC00675 in BC.

### LINC00675 directly interacts with miR-513b-5p

**Figure 3.**
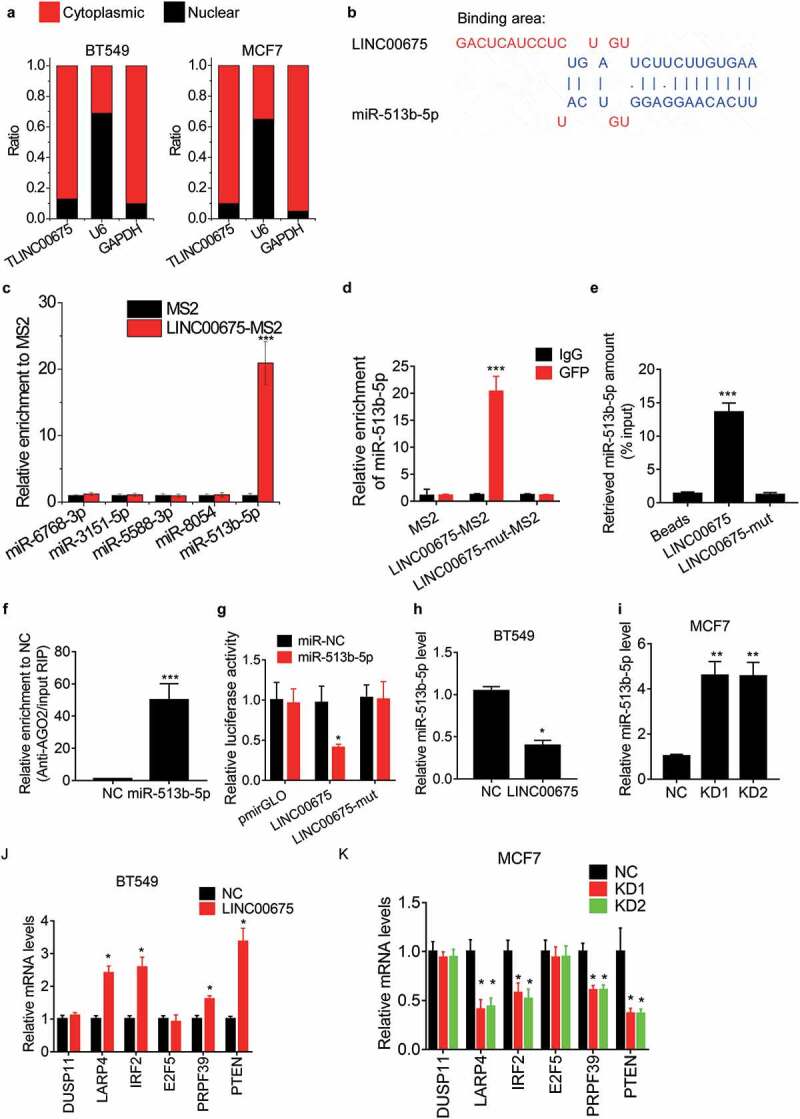
LINC00675 associates with miR-513b-5p

To investigate the underlying mechanism of LINC00675 inhibiting the malignant phenotypes of BC cells, we first detected the cellular distribution of LINC00675. The results of subcellular fractionation showed that LINC00675 mainly located in the cytoplasm of BC cells (Figure 3a). Cytoplasmic lncRNA usually acts as a ceRNA to sponge its target microRNA and exert its regulatory functions [22]. Using an online prediction software LncBase (http://carolina.imis.athena-innovation.gr/diana_tools/web/index.php), we selected the top five microRNAs targeted by LINC00675 for verification. As evidenced by MS2-RIP assay, only miR-513b-5p could be significantly enriched by LINC00675 (Figure 3(c)). LINC00675 contains a binding site for miR-513b-5p (Figure 3(b)). We mutated the binding site of LINC00675 with miR-513b-5p, and found that mutation of LINC00675 failed to associate with miR-513b-5p (Figure 3(d)). The interaction between LINC00675 and miR-513b-5p was also validated by affinity pull-down of endogenous miR-513b-5p using *in vitro* transcribed biotin-labeled LINC00675 (Figure 3(e)). The AGO2-RIP showed that LINC00675 could be significantly enriched by AGO2 by ectopic expression of miR-513b-5p (Figure 3(f)). For further confirmation, the wild-type and mutant LINC00675 were constructed into luciferase reporter plasmid pmirGLO. Transfection of miR-513b-5p mimics significantly decreased the luciferase activity of wild-type reporter, but did not affect the luciferase activity of mutant reporter (Figure 3(g)). Moreover, we found that overexpression of LINC00675 was able to downregulate miR-513b-5p level (Figure 3(h)), whereas knockdown of LINC00675 elevated its expression (Figure 3(i)).

(a) Cytoplasmic and nuclear fractions were extracted from BT549 and MCF7 cells and LINC00675 expression was analyzed by qRT-PCR. The GAPDH mRNA was taken as a cytoplasmic internal reference, while U6 RNA was taken as a nuclear internal reference. (b) The binding sites of LINC00675 with miR-513b-5p were shown. (c) The MS2-RIP assay was performed to detect the indicated microRNA enriched by LINC00675 in BT549 cells. (d) The MS2-RIP followed by qRT-PCR analysis was performed to detect the interaction between LINC00675 and miR-513b-5p in BT549 cells. (e) BT549 cell lysates were incubated with biotin-labeled wild-type or mutant LINC00675; after pull-down, the amount of miR-513b-5p was tested by qRT-PCR. (f) BT549 cells were transfected into miR-513b-5p mimics and its negative control, and then anti-AGO2 RIP was performed to assess the LINC00675 associated with AGO2. (g) Luciferase activity in BT549 cells cotransfected with miR-513b-5p and luciferase reporters containing wild-type or mutant LINC00675. Data are presented as the relative ratio of firefly luciferase activity to renilla luciferase activity. (h) The effect of LINC00675 overexpression on miR-513b-5p was detected using qRT-PCR. (i) The effect of LINC00675 knockdown on miR-513b-5p was detected using qRT-PCR. (j) The expression of the known targets of miR-513b-5p was detected using qRT-PCR in control and LINC00675 overexpressing BT549 cells. (k) The expression of the known targets of miR-513b-5p was detected using qRT-PCR in control and LINC00675 silencing MCF7 cells. *P < 0.05, **P < 0.01, ***P < 0.001.

Previous studies have already identified the targets of miR-513b-5p, such as DUSP11, LARP4, IRF2, E2F5, PRPF39 and PTEN [23–27]. Whether LINC00675 affected the expression of the targets of miR-513b-5p was then assessed. As demonstrated by qRT-PCR experiments, overexpression of LINC00675 significantly upregulated the expression of LARP4, IRF2, PRPF39, and PTEN (Figure 3(j)), whereas knockdown of LINC00675 exerted an opposite function (Figure 3(k)). However, LINC00675 could not influence the expression of DUSP11 and E2F5. All these findings suggest that LINC00675 functions as a ceRNA against miR-513b-5p.

### LINC00675 exerts suppressive function via miR-513b-5p

**Figure 4.**
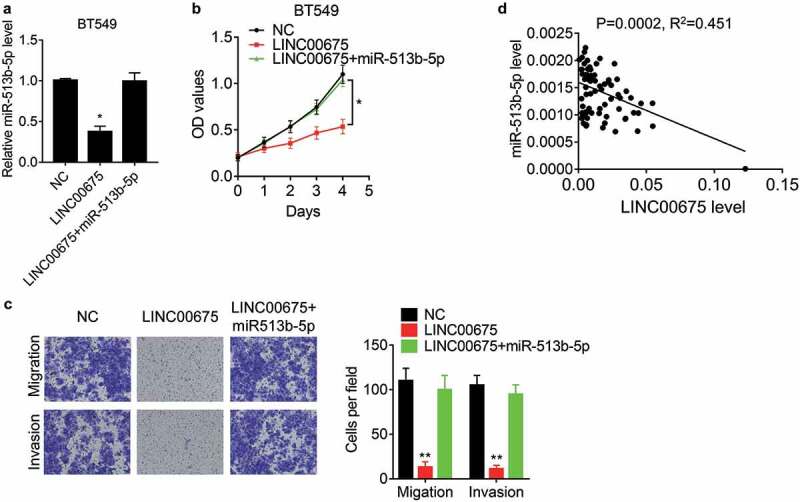
LINC00675 exerts suppressive effects via miR-513b-5p

miR-513b-5p exerts oncogenic or suppressive function in different cancers [23–27], but its effect on BC cells remains unknown. To elucidate whether LINC00675 functions via miR-513b-5p, rescue experiments were carried out. It was found that restoration of miR-513b-5p expression overcame the LINC00675-mediated suppression in BT549 cell proliferation, migration, and invasion (Figure 4(a-c)). Moreover, the miR-513b-5p was detected in BC tissues and showed a negative correlation between LINC00675 and miR-513b-5p (Figure 4(d)), supporting that miR-513b-5p is a bonafide downstream target of LINC00675.

(a) miR-513b-5p mimics were transfected into BT549 cells overexpressing LINC00675, and then the miR-513b-5p expression was examined by qRT-PCR. (b) miR-513b-5p mimics were transfected into BT549 cells overexpressing LINC00675, and then the cell proliferation was assessed by CCK-8 assay. (c) miR-513b-5p mimics were transfected into BT549 cells overexpressing LINC00675, and then the cell migration and invasion was assessed by transwell assay. The represent images (left) and statistical result (right) was shown. The cells were counted in 10 random field under microscope. (d) The Pearson correlation analysis between LINC00675 and miR-513b-5p expression in 74 BC tissue samples. *P < 0.05, **P < 0.01.

### METTL3-mediated m^6^A modification of LINC00675 regulates its ceRNA activity

**Figure 5.**
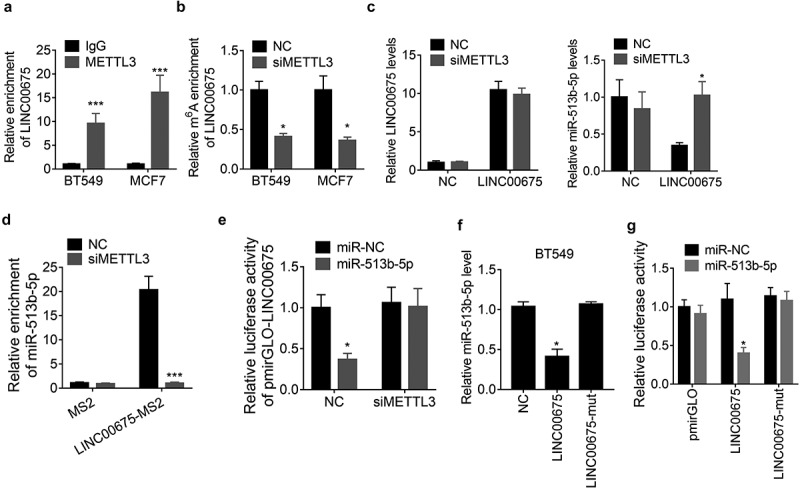
m^6^A modification is involved in the ceRNA activity of LINC00675

A recent study reported that N^6^-Methyladenosine (m^6^A) modification contributes to the ceRNA activity of lncRNA [16]. To elucidate whether LINC00675 regulated miR-513b-5p in this manner, we performed a RIP assay to detect the association of LINC00675 with METTL3, the core component of m^6^A methylase complex, and observed that LINC00675 could be significantly pulled down by anti-METTL3 antibody compared to IgG (Figure 5(a)). Moreover, knockdown of METTL3 resulted in a decrease of LINC00675 m^6^A level as demonstrated by MeRIP assay (Figure 5(b)). However, knockdown of METTL3 did not affect the LINC00675 expression, but abolished the downregulation of miR-513b-5p expression induced by LINC00675 in BT549 cells (Figure 5(c)), suggesting that METTL3 may influence ceRNA activity of LINC00675. To validate this hypothesis, we performed a MS2-RIP assay and found that depletion of METTL3 expression, LINC00675 failed to associate with miR-513b-5p (Figure 5(d)). Furthermore, BT549 cells were co-transfected with the pmirGLO-LINC00675 reporter, METTL3 siRNAs and miR-513b-5p mimics. Similarly, the results of luciferase reporter experiment demonstrated that the suppressive effect of miR-513b-5p on pmirGLO-LINC00675 was abolished by deletion of METTL3 (Figure 5(e)). The m^6^A sites of LINC00675 was predicted using the SRAMP online tool (http://www.cuilab.cn/sramp/) and the results showed that only one m^6^A site was predicted in LINC00675 transcript (Supplemental Figure 1). We mutated this site (A to G) and found that this mutation did not affect the miR-513b-5p expression (Figure 5(f)). Moreover, miR-513b-5p was unable to decrease the activity of this mutant LINC00675 reporter (Figure 5(g)), suggesting the importance of m^6^A sites of LINC00675 in regulating the expression of miR-513b-5p and its interaction with miR-513b-5p. Collectively, these findings suggest that m^6^A modification is critical for LINC00675 sponging miR-513b-5p.

(a) The RIP assay using IgG or METTL3 antibody was carried out to detect the LINC00675 pulled down by METTL3 in BT549 and MCF7 cells. (b) The MeRIP analysis of the m6A level of LINC00675 in BT549 and MCF7 cells transfected with METTL3 siRNAs. (c) The qRT-PCR analysis of the LINC00675 and miR-513b-5p levels in control and LINC00675 overexpressing BT549 and MCF7 cells transfected with METTL3 siRNAs. (d) The MS2-RIP analysis of the interaction between LINC00675 and miR-513b-5p in BT549 cells transfected with METTL3 siRNAs. (e) The luciferase reporter analysis of the LINC00675 reporter in BT549 cells cotransfected with miR-513b-5p mimics and METTL3 siRNAs. (f) The effect of wild-type or mutant LINC00675 overexpression (mutation in m6A site) on miR-513b-5p was detected by qRT-PCR. (g) Luciferase activity in BT549 cells co-transfected with miR-513b-5p and luciferase reporters containing wild-type or mutant LINC00675 (mutation in m6A site). Data are presented as the relative ratio of firefly luciferase activity to Renilla luciferase activity. *P < 0.05, ***P < 0.001.

## Discussion

Here, we identified LINC00675 as a tumor-suppressive lncRNA significantly downregulated in BC. Gain- and loss-of-function experiments demonstrated that overexpression of LINC00675 attenuated the proliferation, migration, and invasion ability of BC cells, while knockdown of LINC00675 enhanced these cellular phenotypes. Moreover, decrease of LINC00675 expression was associated with advanced tumor grade and lymphovascular invasion. BC patients with high LINC00675 level exhibited better clinical outcome than those with low LINC00675 expression. Therefore, LINC00675 may serve as a prognostic factor for BC patients.

Previous studies have implicated the opposite function of LINC00675 in different specific types of cancer via diverse mechanism. For instance, in gastric cancer, LINC00675 was found to be downregulated in cancer tissues and inhibit metastasis through associating with vimentin and enhancing its phosphorylation [11]. LINC00675 also competitively binds with LSD1 and promotes the binding of LSD1 and its target H3K4me2, thus suppressing SPRY4 transcription [28]. Xu et al. reported that LINC00675 inhibits tumorigenesis and metastasis via suppressing Wnt/β-catenin signaling in esophageal squamous cell carcinoma [14]. The same mechanism of LINC00675 was also found in cervical cancer and colorectal cancer [12,29]. In other cases, LINC00675 has been identified as oncogene. LINC00675 is upregulated in androgen-insensitive prostate cancer cell lines and castration-resistant prostate cancer patients. LINC00675 directly modulates androgen receptor’s (AR) interaction with MDM2 and blocks AR’s ubiquitination and stabilizes GATA2 mRNA which is a co-activator in the AR signaling pathway [15]. In glioma, high-expression of LINC00675 was an independent unfavorable prognostic predictor. LINC00675 facilitates glioma cell proliferation, migration and invasion through regulating TRIP6 [13]. Here, we verified that LINC00675 acted as a ceRNA against miR-513b-5p, suppressing BC cell proliferation, migration and invasion. Previous studies have reported that miR-513b-5p is involved in the proliferation, metastasis and drug resistance of cancer cells. The targets of miR-513b-5p includes DUSP11, LARP4, IRF2, E2F5, PRPF39, and PTEN [23–27]. Here, our date demonstrated that LINC00675 significantly upregulated the expression of LARP4, IRF2, PRPF39 and PTEN, but could not influence the expression of DUSP11 and E2F5. LARP4 is an RNA binding protein which is involved in T cell activation-dependent mRNA stabilization [30]. LARP4 also functions as a suppressor for motility of ovarian cancer cells [31]. IRF2 upregulates β-catenin expression to modulate cellular survival [32]. Loss of IRF2 leads to immune evasion through decreased MHC class I antigen presentation and increased PD-L1 expression in cancers [33]. PRPF39 is a regulator of cisplatin sensitivity in liver cancer [23]. PTEN is a well-known tumor suppressor. The loss of PTEN activity, identified in a series of primary and metastatic tumors, such as breast cancer, leads into uncontrolled transduction of the PI3K signal which is involved in a series of biological processes, such as cellular motility, invasion, proliferation, and survival [34]. These findings suggested that LINC00675- miR-513b-5p may exerted different function via regulating specific target genes.

m^6^A modification is the most abundant eukaryotic RNA modification and involved in regulating essential biological processes of the transcriptome, such as RNA splicing, decay, nuclear export, translation, and microRNA processing [17]. Dysregulation of the m^6^A regulators has increasingly been found in many neoplasms, including BC. For example, METTL3 upregulates the expression of onco-protein HBXIP via m^6^a modification, which promotes BC progression [35]. METTL3 increases m^6^A level of the ITGA6 mRNA 3ʹUTR and promotes the translation of ITGA6 mRNA via binding of the m^6^A readers YTHDF1 and YTHDF3, inducing BC growth and metastasis [36]. m^6^A demethylase FTO also promotes BC development via decreasing the m^6^A level of BNIP3 mRNA and inducing its degradation in an YTHDF2 independent manner [37]. Recently, m^6^A modification has been found to regulate the ceRNA activity of lncRNA. linc1281 ensures embryonic stem cells identity by sponging let-7 family, and this lncRNA-microRNA interaction is regulated by m^6^A modification [16]. Similarly, our present study revealed that METTL3 associated with LINC00675 and increased its m^6^A level, which did not affect the LINC00675 expression. Knockdown of METTL3 significantly attenuated the interaction between LINC00675 and miR-513b-5p. These findings suggest the importance of m^6^A modification of lncRNA in regulating microRNA.

## Conclusions

**Figure 6.**
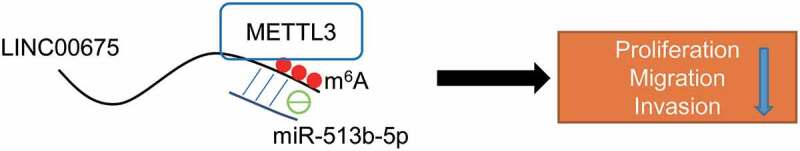
The schematic diagram of proposed mechanism of LINC00675 regulating miR-513b-5p in BC cells

In summary, our study reveals that LINC00675 is downregulated in BC, and inhibits BC cell proliferation, migration and invasion by acting as a ceRNA to regulate miR-513b-5p, which is regulated by METTL3-mediated m^6^A modification (Figure 6).

## Supplementary Material

Supplemental Material

## Data Availability

The datasets used during this research are available.
