# Supplementary material for: N6-Methyladenosine-regulated LINC00675 suppress the proliferation, migration and invasion of breast cancer cells via inhibiting miR-513b-5p
Source: Bioengineered. 2021 Dec 1;12(2):10690–702. doi: 10.1080/21655979.2021.2001905 (PMC8810037; doi:10.1080/21655979.2021.2001905)
Supplement: Supplemental Material [file KBIE_A_2001905_SM4590.zip › supplementary/Supplemental_figure_legend.docx]

**Supplemental Figure 1. m^6^A modification is involved in the ceRNA activity of LINC00675.** The m^6^A sites of LINC00675 was predicted using the SRAMP online tool.
